# Synthesis, Characterization, and Therapeutic Efficacy of ^177^Lu-DMSA@SPIONs in Nanobrachytherapy of Solid Tumors

**DOI:** 10.3390/pharmaceutics15071943

**Published:** 2023-07-13

**Authors:** Dragana Stanković, Magdalena Radović, Aljoša Stanković, Marija Mirković, Aleksandar Vukadinović, Milica Mijović, Zorana Milanović, Miloš Ognjanović, Drina Janković, Bratislav Antić, Sanja Vranješ-Đurić, Miroslav Savić, Željko Prijović

**Affiliations:** 1 Vinča Institute of Nuclear Sciences—National Institute of the Republic of Serbia, University of Belgrade, 11001 Belgrade, Serbia; dragana.s@vin.bg.ac.rs (D.S.); magdalena.lazarevic@vin.bg.ac.rs (M.R.); mmarija@vin.bg.ac.rs (M.M.); vukadinovic@vin.bg.ac.rs (A.V.); zorana.milanovic@vin.bg.ac.rs (Z.M.); miloso@vin.bg.ac.rs (M.O.); drinaj@vin.bg.ac.rs (D.J.); bantic@vin.bg.ac.rs (B.A.); sanjav@vin.bg.ac.rs (S.V.-Đ.); 2 University Clinical Centre of the Republic of Srpska, 78000 Banja Luka, Bosnia and Herzegovina; aljosa.stankovic@kc-bl.com; 3 Faculty of Medicine, Institute of Pathology, University of Priština in Kosovska Mitrovica, 38220 Kosovska Mitrovica, Serbia; milica.mijovic@med.pr.ac.rs; 4 Faculty of Pharmacy, University of Belgrade, 11000 Belgrade, Serbia; miroslav@pharmacy.bg.ac.rs

**Keywords:** nanobrachytherapy, nanoparticles, ^177^Lu, radionuclide therapy, tumor

## Abstract

As an alternative to classical brachytherapy, intratumoral injection of radionuclide-labeled nanoparticles (nanobrachytherapy, NBT) has been investigated as a superior delivery method over an intravenous route for radionuclide therapy of solid tumors. We created superparamagnetic iron oxide nanoparticles (SPIONs) coated with meso-1,2-dimercaptosuccinic acid (DMSA) and radiolabeled with Lutetium-177 (^177^Lu), generating ^177^Lu-DMSA@SPIONs as a potential antitumor agent for nanobrachytherapy. Efficient radiolabeling of DMSA@SPIONS by ^177^Lu resulted in a stable bond with minimal leakage in vitro. After an intratumoral injection to mouse colorectal CT-26 or breast 4T1 subcutaneous tumors, the nanoparticles remained well localized at the injection site for weeks, with limited leakage. The dose of 3.70 MBq/100 µg/50 µL of ^177^Lu-DMSA@SPIONs applied intratumorally resulted in a high therapeutic efficacy, without signs of general toxicity. A decreased dose of 1.85 MBq/100 µg/50 µL still retained therapeutic efficacy, while an increased dose of 9.25 MBq/100 µg/50 µL did not significantly benefit the therapy. Histopathology analysis revealed that the ^177^Lu-DMSA@SPIONs act within a limited range around the injection site, which explains the good therapeutic efficacy achieved by a single administration of a relatively low dose without the need for increased or repeated dosing. Overall, ^177^Lu-DMSA@SPIONs are safe and potent agents suitable for intra-tumoral administration for localized tumor radionuclide therapy.

## 1. Introduction

One of the most critical issues in curing cancer with nanotherapy is the delivery of the nanomaterial into the tumor tissue at a level sufficient to achieve therapeutic effects. About 50 nanoformulations for medical applications with significant market success have already been approved by the US Food and Drug Administration-FDA [1,2,3,4,5,6,7]. These mainly refer to liposomal drug formulations designed to reduce toxicity and improve the pharmacokinetic profile of chemotherapeutic drugs.

An intravenous administration is usually the most desirable way to deliver nanomaterials because such a way of delivery ensures that the material can reach tumors in places inaccessible by surgery as well as their metastases. However, previous studies have shown that the percent of injected dose (%ID) of nanoparticles delivered to solid tumors after intravenous (i.v.) administration is very low, with a median of 0.7–2.24% [8,9], and the percentage delivered to targeted cancer cells is even lower (0.0014% ID) [10]. It was found that the size and properties of nanoparticles and the pathophysiology of the tumor itself greatly influence the nanoparticle biodistribution after i.v. application in mice [11,12,13,14,15,16].

Investigations on mice showed that after i.v. injection, nanoparticles are primarily cleared by the liver and spleen, reducing the nanoparticles concentration available to the tumor [17,18]. Consequently, if radiolabeled nanoparticles are used, liver damage would occur rather than tumor destruction. Tavares et al. have tried to solve this delivery problem by removing the liver’s Kupffer cells, but delivery efficiency was not elevated to more than 2% regardless of the size and composition of the nanomaterial as well as the type of tumor [19]. Ouyang et al. have found that to improve nanoparticle delivery to the tumor, the dose threshold should be one trillion nanoparticles. Doses above this threshold overload Kupffer cell uptake rates, nonlinearly reduce their clearance from the liver, prolong their circulation time, and thus improve their tumor delivery efficiency to 12 %ID [20]. 

Considering the aforementioned problems, many researchers have been investigating the therapeutic effect of radiolabeled nanoparticles on mouse tumors after direct intratumoral injection (nanobrachytherapy, NBT). It is believed that by using this approach, a sufficient amount of nanomaterials in the tumor tissue would be provided and that the required radiation dose similar to that of radiation brachytherapy would be delivered [21,22,23]. Although this method of administration has a limited number of indications, it is still of clinical importance due to the high incidence of cases in which it is applicable.

The latest research in the field of nuclear medicine confirms that nanoparticles have an enormous potential for application in NBT [24,25]. Nanoparticles based on gold, silica, or dendrimers and radiolabeled with ^177^Lu, are in the spotlight of the theranostic approach for the treatment of tumors [25,26,27]. The radionuclide ^177^Lu has received considerable attention due to its availability and favorable emission characteristics. The ^177^Lu is a radionuclide that has a physical half-life of 6.7 days and emits beta/gamma radiation. The maximum energy of beta particles is 0.497 MeV, and the maximum range in the tissue is about 2 mm. These properties enable the ‘cross fire’ effect on the adjacent cancer cells but a minimal effect on the normal surrounding tissue [28,29,30]. In addition, ^177^Lu has two gamma emissions at 113 keV and 208 keV that allow SPECT imaging.

We synthesized superparamagnetic iron oxide nanoparticles (SPIONs) coated with DMSA and radiolabeled with ^177^Lu to generate ^177^Lu-DMSA@SPIONs, a nanoplatform suitable for NBT. Such designed radiolabeled coated iron-oxide-based nanoparticles were characterized for size, shape, colloidal stability, radiolabeling efficiency, and stability in vitro. Further, the in vivo behavior of the designed nanoplatform was tested on mouse colorectal (CT-26) and breast (4T1) tumor-bearing mice to determine the pharmacokinetic behavior. The radiolabeled nanoplatform was tested in CT-26 and 4T1 tumor models, varying the number of treatments and the radiation doses. The intratumoral distribution, effects on the cancer tissue at the cellular level, and antitumor efficacy were observed together with the body mass as the signs of general toxicity. The goal was to decipher the mode of action and estimate the suitability of the nanoplatform for nanobrachytherapy of inoperable or hard-to-reach solid tumors. 

## 2. Materials and Methods

### 2.1. Materials

Ferric chloride hexahydrate (FeCl_3_·6H_2_O), ferrous sulfate heptahydrate (FeSO_4_·7H_2_O), ammonium hydroxide (≥25% NH_3_), sodium hydroxide (NaOH), and chloric acid (HCl) were purchased from Sigma-Aldrich (Munich, Germany). ITG Isotope Technologies Garching GmbH (Munich, Germany) is a supplier of ^177^LuCl_3_ solution whose specific activity was >500 GBq/mg Lu. The rest of the reagents used in this study are bought from Sigma-Aldrich Co (St. Louis, MO, USA). Mouse cancer cell lines: CT-26 (ATCC^®^ CRL-2638^™^) and 4T1 (ATCC^®^ CRL-2539™) were bought from the American Type Culture Collection (ATCC, Rockville, MD, USA).

### 2.2. Preparation of Bare SPIONs

The synthesis of bare SPIONs was performed using the co-precipitation method developed by Massart [31]. The solution of Fe ions was prepared by consecutively dissolving ferrous sulfate (0.625 g) and ferric chlorides (1.216 g) in a 1:2 FeSO_4_∙7H_2_O: FeCl_3_·6H_2_O molar ratio in 30 mL of deionized and degassed water with stirring. When the temperature reached about 50 °C, approximately 20–25 mL of 25% NH_4_OH solution was added drop by drop until pH = 10–11 of the solution was achieved. The constant stirring was continued for another 1 h while the temperature was maintained at 80 °C. The sample was then allowed to cool down to ambient temperature. The obtained black precipitate of Fe_3_O_4_ (SPIONs) was separated from the supernatant using magnetic decantation, in which strong external magnet was used, and the supernatant was discarded. The precipitate was then washed several times with distilled water until the pH of the supernatant was neutral. After that, the SPIONs were dispersed in 10 mL of ultrapure (Milli-Q) water.

### 2.3. Functionalization with Meso-2,3-dimercaptosuccinic Acid (DMSA)

To coat the SPIONs with DMSA (DMSA@SPIONs), 10 mL of 80 mM solution of DMSA in dimethylsulfoxide (DMSO) was added to a suspension of SPIONs (~150 mg) (pH~6). The suspension was then stirred at ambient temperature for 24 h, followed by washing by overnight dialysis (12 kDa MWCO) against deionized water (changing water three times), and the final pH was 7–8.

### 2.4. Characterization of DMSA-SPIONs

A transmission electron microscope JEOL-TEM 1010 (JEOL, Tokyo, Japan), with an acceleration voltage up to 100 kV was used to examine the morphology of synthesized and uncoated SPIONs. After dilution of SPIONs samples, they were placed on a carbon-coated 200 mesh copper grid and naturally dried at ambient temperature. Using Image J software for analysis of obtained electron micrographs, the average diameter of SPIONs was determined (*d_TEM_*). Around 350 nanoparticles were randomly observed. In order to determine the mean size ($\bar{x}$), the standard deviation (σ), and the index of polydispersity (PDI%), the data were fitted to a log-normal function ($y=y_{0}+\frac{A}{\sqrt{2\pi }\;\omega x}\exp\frac{-{\left[\ln\frac{x}{x_{c}}\right]}^{2}}{2\omega^{2}}$).

The XRD powder method was applied for the crystallographic analysis of the synthesized bare SPIONs. Diffractometer SmartLab^®^ X-ray (Rigaku, Tokyo, Japan) equipped with cobalt cathode (CuKαradiation), was used for recording the high-resolution diffraction patterns. For acceleration, the voltage of 40 kV and current of 30 mA was applied. Sample preparation was performed with a zero-background silicon wafer by flatting out a dried powder. The data were collected using a continuous scan mode (2 deg/min) to collect 2𝜃 data from 10 to 70 degrees. Using Scherrer’s equation, the mean crystallite size, *d_XRD_*, was determined ($d_{XRD}=\frac{K\cdot \lambda }{\beta \cdot \cos\theta }$).

A Nano ZS90 (Malvern, UK) apparatus with a 4 mW He-Ne laser source (λ = 633 nm) was used for DLS measurement of particle size distributions of bare SPIONs and DMSA-coated SPIONs. The same apparatus was utilized for zeta potential measurements of bare SPIONs and DMSA@SPIONs samples in single-use zeta cells (DTS 1070) at 25.0 ± 0.1 °C, at pH = 7, and at *I* = 0.01 M. 

The analysis of chemical groups on the surface of bare SPIONs and DMSA@SPIONs was performed using a Nicolet iS50 FT-IR Thermo Fisher Scientific spectrophotometer at ambient temperature in the region from 400 cm^−1^ to 4000 cm^−1^.

### 2.5. Radiolabeling of DMSA@SPIONs with ^177^Lu

1.1 mL of DMSA@SPIONs (~10 mg) was incubated by stirring for 1 h with 10 μL of ^177^LuCl_3_ solution (~370 MBq) at ambient temperature. In total, 0.1 M NaOH was used to adjust the pH of the suspension to 6.5. After incubation, magnetic decantation was applied for the separation of radiolabeled DMSA@SPIONs from unbound ^177^Lu^3+^. The supernatant with unbound ^177^Lu^3+^ was discarded, while the remaining precipitate was rinsed out with deionized water several times. Labeling yield is derived from the ratio of the ^177^Lu-DMSA@SPIONs—activity measured after magnetic separation and the total initial radiolabeling activity. The normal saline solution was used for the dilution of radiolabeled DMSA@SPIONs up to a final volume of 4.0 mL. Instant thin-layer chromatography (ITLC/SG), using 0.9% NaCl as the mobile phase, was used to determine the radiochemical purity of ^177^Lu-DMSA@SPIONs. They remain at the origin (*Rf* = 0.0), while free ^177^Lu^3+^ goes up with the solvent front (*Rf* = 0.9–1.0). For the radioactivity measurement of samples, a dose calibrator CRC-15 beta radioisotope (Capintec, Florham Park, NJ, USA) and NaI (Tl) gamma counter (Wizard 2480, Perkin Elmer, Waltham, MA, USA) were used. The same geometric conditions were applied for all measurements. The obtained results are presented as the mean ± standard deviation.

### 2.6. In Vitro Stability Studies

For the ^177^Lu-DMSA@SPIONs stability at ambient temperature, the original suspension was left for the 144-h period. The in vitro stability of ^177^Lu-DMSA@SPIONs was checked by incubating the quantity of 100 µL ^177^Lu-DMSA@SPIONs in 900 µL of normal saline or human serum (HS, National Blood Transfusion Institute, Serbia) over 144 h incubation time at 37 °C. At certain time intervals (2, 12, 24, 72, 96, and 144 h), samples were taken, and the radiochemical purity of ^177^Lu-DMSA@SPIONs was determined using ITLC chromatography as previously described.

### 2.7. Cell Lines

The mouse CT-26 colorectal carcinoma and mouse 4T1 mammary gland tumor cell lines were thawed from a frozen stock at 37 °C briefly and diluted with 8 mL cold RPMI-1640 medium, spun down at 600× *g* for 4 min and cultured with 12 mL full RPMI-1640 medium with addition of 10% (*v*/*v*) FBS, 100 IU/mL penicillin and 100 µg/mL streptomycin. An incubator at 37 °C with CO_2_-humidified air (5%) was used for cell maintenance. After growing cells in monolayer culture to 80% confluence, they were harvested using trypsin and washed with PBS twice. After re-suspension in RPMI and counting in a Neubauer chamber, the cell density was adjusted to 1 × 10^7^ cells/mL for CT-26 and 4 × 10^6^ cells/mL for 4T1 cells.

### 2.8. Mouse Tumor-Bearing Models

The Institute for Biological Research “Siniša Stanković”, Belgrade, Serbia, provided the experimental rodents (adult female BALB/c mice, 8–10 weeks old). For the housing of mice, a P/N ventilated IVC cage system (Allentown, PA, USA) was used. The temperature (22 ± 2 °C) and humidity (45–50%) were constant. Housing conditions included the following: artificial lighting environment (12 h light /12 h dark cycle), free access to food and water (commercial pellet food and tap water), and the 4 animals, each in polypropylene cages with wood chips bedding. The adaptation period for mice was one week before starting the experiments. The Ethical Committee of the “Vinča” Institute of Nuclear Sciences, Belgrade, gave a positive opinion, and the Ministry of Agriculture, Forestry and Water Economy of the Republic of Serbia, in accordance with the National Law on Animal Welfare and EU Directive 2010/63/EU, issued a permit (No. 323-07-10153/2022-05) for these experiments on mice.

The animals were weighed twice before grouping. Mouse tumor xenografts model were induced by a single subcutaneous injection of CT-26 at 1 × 10^6^/100 µL and 4T1 at 4 × 10^5^/100 µL into the shaved loins. Tumor growth was monitored every two days. Body weights were measured two days before dosing, as well as every two days (immediately before tumor size measurement) in all the groups. Tumors size was measured in three dimensions using a digital caliper, and tumor volume was calculated using the following formula: *V* (mm^3^) = (*length* × *width* × *height*)/2.

At the end of the experiment, the mean body weight for each group was calculated. The experiment was stopped on day 14 by cervical dislocation of the mice or earlier if the tumor reached a size of ≥2500 mm^3^ or caused excessive pain or distress to the animal. 

### 2.9. Pharmacokinetics and Biodistribution In Vivo

To estimate general suitability of the ***^177^Lu-DMSA@SPIONs*** for NBT, a group of three mice with large (initial tumor size ~300 mm^3^) CT-26 tumors were injected ***intratumorally*** in the dose of 3.70 MBq/100 μg/50 μL of vehicle. At predetermined time points during time course of 11 days, the animals were anesthetized by intraperitoneal injection of ketamine/xylazine (90/10 mg/kg) and in vivo imaged using a BRUKER^®^ In-Vivo Xtreme II device (Billerica, MA, USA). Images were captured in a single lateral imaging position, using an appropriate filter to convert the radiation to a light that was recorded by an onboard CCD camera. The produced photos were used to quantitate the level of radiation remaining in the tumors in live animals during the time course to generate the pharmacokinetic profile. At the end of the experiment, the animals were sacrificed by cervical dislocation, organs of interest removed, arranged on a plastic holder, and imaged as for the whole animals to demonstrate presence or absence of radioactivity. 

To more precisely determine the biodistribution after intratumoral injection, groups consisting of 5–7 mice with CT26 or 4T1 tumors (125 ± 50 mm^3^) were formed. Groups received ^177^Lu-DMSA@SPION at dose of 3.70 MBq/100 μg/50 μL and get sacrificed after 24 h and at the termination of the study. Before imaging and sacrifice, mice were anesthetized using ketamine/xylazine (90/10 mg/kg) injection intraperitoneally. After sacrifice by cervical dislocation, organs of interest and tumors were weighed, and their radioactivity was measured on a gamma counter (Wizard 2480, Perkin Elmer, USA). Obtained results were presented as a ratio of the activity of the organs and the total injected dose (%ID/organ). 

### 2.10. The Therapeutic Efficacy of ^177^Lu-DMSA@SPIONs

To estimate the therapeutic efficacy of ^177^Lu-DMSA@SPIONs against CT-26 and 4T1 tumors, random groups, each containing 5–7 tumor-bearing mice, were formed. Therapy groups were treated by an intratumoral injection of 3.70 MBq/100 μg of ^177^Lu-DMSA@SPIONs in 50 μL of vehicle per 100 mm^3^ of tumor at day 0 as a single dose or at days 0 and 5 as repeated dose regime. As a control, one group received no treatment while another group received non-radioactive DMSA@SPIONs at the same regime as the therapy groups.

Further, another therapy regime to treat CT-26 and 4T1 tumors included varying the doses at 1.85 and 9.25 MBq/100 μg of ^177^Lu-DMSA@SPIONs in 50 μL of vehicle per 100 mm^3^ of tumor volume in a single dose was tested. The mice’s well-being was monitored daily, while the tumor size and the mice’s body mass (as an indicator of systemic toxicity) were recorded every second day for two weeks. At the end of the follow-up, the animals were sacrificed by cervical dislocation, and tumors and organs of interest from all groups were removed and accurately weighted. The representative tumors and organs were further processed for histopathology analysis.

### 2.11. Histopathology Analysis

At the end of the therapeutic efficacy study and after tumor removal, the Tissue-Tek^®^ O.C.T. medium was used to embed the tumor tissue and organs of interest, and snap-freeze them for further histopathological analysis. Each frozen tissue was cut using a rotary cryotome (Kedi Instrumental Equipment, Jiahua, China) to obtain 5 and 8 μm thick slides. The 5 μm slides were stained with hematoxylin and eosin staining (H&E), dehydrated by xylene replacement solvent, and mounted in Entellan for routine histopathology analysis, while 8 μm sections were stained by Prussian blue (PB) and counterstained by Nuclear Fast Red, mounted in ImmunoHistoMount for the demonstration of the nanomaterial distribution. 

According to tumor morphology, all slides were interpreted qualitatively. Semi-quantitative analysis based on the degree of necrosis in relation to the tumor volume (discrete—u < 1/3; moderate—u from 1/3 to 2/3; abundant—u > 2/3 of the tumor volume) was used to interpret the slides. The slides were examined under a microscope (Zeiss AxioVert A1) using 200- or 400-times magnification. All H&E and PB slides were reviewed by two independent experienced pathologists blinded for other data. The original photomicrographs were captured using a Zeiss Axiocam digital color camera (Carl Zeiss, Oberkochen, Germany).

### 2.12. Statistical Analysis

Results were analyzed using IBM^®^ SPSS^®^ Statistics version 25 software (IBM, Armonk, NY, USA) and were presented as mean ± standard deviation (SD). Statistical significance was examined using one-way ANOVA with a post hoc least significant difference (LSD) test. The difference is considered significant at *p* < 0.05.

## 3. Results and Discussion

### 3.1. Characterization of SPIONs and DMSA@SPIONs

**Figure 1:**
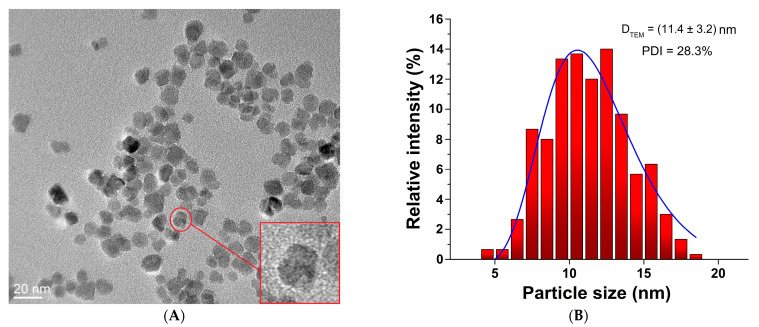
(**A**) TEM images of uncoated SPIONs prepared by co-precipitation method, (**B**) particle size distribution.

After synthesis by the chemical co-precipitation method, the size and morphology of uncoated SPIONs were examined by transmission electron microscopy (TEM). The TEM micrograph shows that the nanoparticles are spherical (“pseudo sphere”) with an average diameter of 11.4 ± 3.2 nm and a PDI of 28.3% (Figure 1). The PDI was used as an indicator for nanoparticle stability and uniformity of formation. This higher value of PDI indicates lower particle stability and the tendency towards the aggregation of SPIONs.

**Figure 2:**
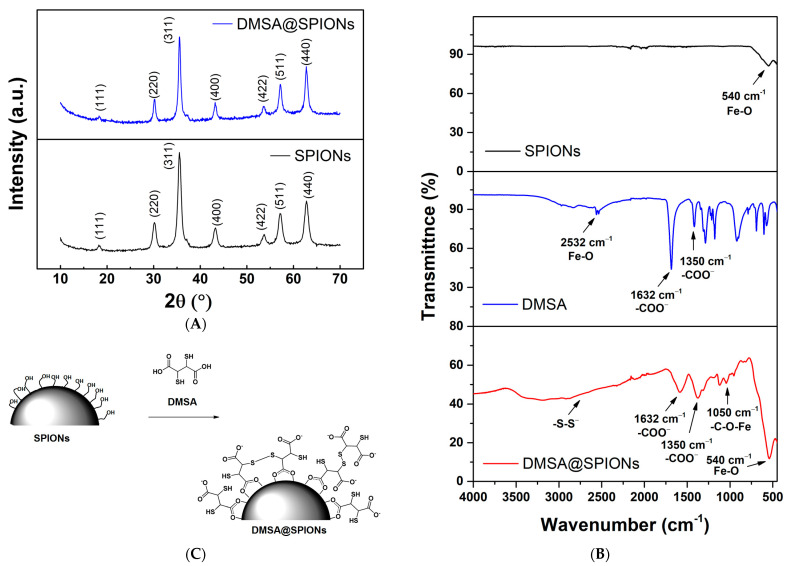
(**A**) XRD curves of SPIONs and DMSA@SPIONs, (**B**) FT-IR spectra of SPIONs, DMSA, and DMSA@SPIONs, (**C**) schematic diagram of SPIONs coating with DMSA.

The XRD patterns of the uncoated SPIONs and DMSA@SPIONs show well-defined peaks, which clearly indicate that the samples are crystalline (Figure 2A). The patterns of both samples revealed the following characteristic reflections of the spinel structure: (111), (220), (440), and (311), which unambiguously coincide with the spinel structure attributed to magnetite (Fe_3_O_4_) (JCPDS # 19-0629), but also maghemite (γ-Fe_2_O_3_) (JCPDS # 110614). However, it is known that it is difficult to notice the difference between the phases of magnetite (space group Fd-3m) and maghemite (space group P4332) in the sample. In accordance with the data from the literature and the observed XRD patterns, it can be concluded that the synthesized SPIONs are most likely a mixture of two phases, magnetite and maghemite [32]. The determined mean crystallite size based on the most intensive (311) reflection at 2θ~36° was 12.2 nm, which is consistent with the average diameter (DTEM) of the nanoparticles calculated by the TEM method 11.4 ± 3.2 nm.

The size of uncoated or DMSA-coated SPIONs was also determined by the DLS method. The mean value of the nanoparticle diameters obtained by DLS measurements, DH, presents the spherical hydrodynamic diameter consisting of the particle and the hydrated coating together. The hydrodynamic diameter value for SPIONs was 46.1 ± 3.8 nm. The sizes of particles based on TEM measurement were significantly smaller in comparison with those achieved by the DLS method (*p* < 0.05). This can probably be attributed to the fact that aggregates of SPIONs are already present in the suspension due to their lower stability. The influence of the coating on the value of the SPIONs hydrodynamic diameter was investigated by DLS measuring DMSA-coated SPIONs. Their mean values of 140.3 ± 6.5 nm showed that the hydrodynamic diameter of nanoparticles increases during SPIONs coating (*p* < 0.05) as it was expected.

The zeta (ζ) potential is the measure of the stability of the nanoparticles in the suspension. If the electric charge on the surface of the nanoparticles is higher, they will be safer from being agglomerated in the buffer solution due to the strong repulsive forces among particles [33]. The zeta potential of bare SPIONs was +12.5 mV, which changed to −35.1 mV for DMSA@SPIONs due to the presence of numerous surface carboxyl and thiol functional groups, additionally showing their increased stability in the suspension if compared to uncoated SPIONs.

The surface presence of DMSA on the SPIONs was also confirmed by FT-IR analysis (Figure 2B). DMSA@SPIONs clearly show band patterns corresponding to all constituents. The characteristic peaks originating from the stretching of the C-O-Fe bond present in the DMSA@SPIONs spectrum at 1050 cm^−1^ are confirmed because DMSA molecules bind to the SPIONs surface [34]. Also, as confirmation of the coating SPIONs with DMSA, there are peaks present at 1632 and 1350 cm^−1^ related to the symmetric and asymmetric stretching of carboxyl groups (-COO^−^) on the DMSA@SPIONs surface. The broad band at 2532 cm^−1^ in the DMSA spectrum belongs to the stretching vibrational of the S-H bond in the thiol group.

The reason for its absence in the DMSA@SPIONs spectrum is the oxidation of -SH groups to disulfide -S-S- groups that absorb in the lower IR region so that their presence would be proven by Raman spectroscopy [35]. The peak at 540 cm^−1^ that occurs in bare SPIONs and DMSA@SPIONs spectra belongs to the Fe-O stretching vibration. Based on these results, it can be assumed that during the coating of SPIONs with DMSA, the polar Fe-O-C bonds are formed with the separation of water molecules. Also, the free -SH groups of DMSA react with each other to form disulfide -S-S- bonds in the envelope (Figure 2C).

### 3.2. Radiolabeling of DMSA-SPIONs and In Vitro Stability of ^177^Lu-DMSA@SPIONs

DMSA@SPIONs were radiolabeled with ^177^Lu^3+^ using the direct radiolabeling method. Radiolabeling studies found that ^177^Lu-DMSA@SPIONs can be obtained in an acceptable radiolabeling yield (86.6 ± 2.1%), and after separation by magnetic decantation, the radiochemical purity of the ^177^Lu-DMSA@SPIONs, detected by ITLC on SG strips, was >99%.

**Figure 3:**
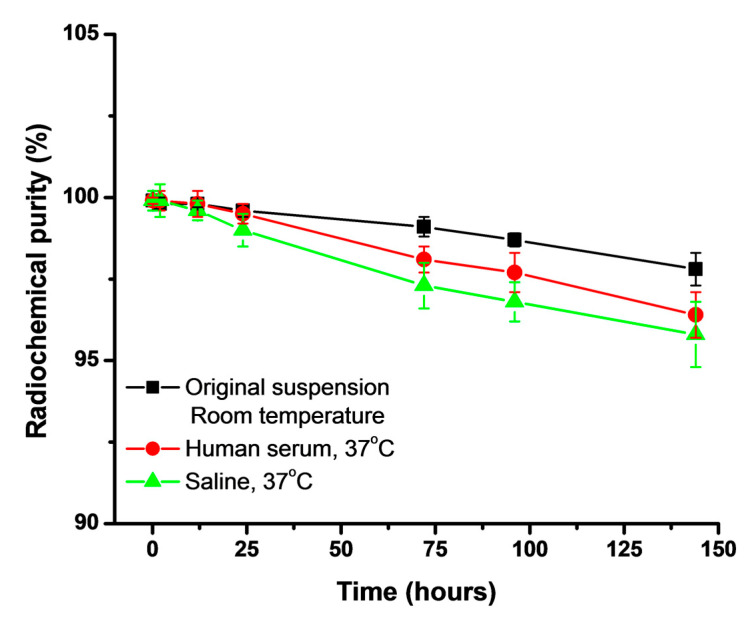
In vitro stability of ^177^Lu-DMSA@SPIONs stored in the original suspension at ambient temperature, incubated in human serum at 37 °C, and saline solution at 37 °C. The values represent mean ± SD, *n* = 3.

The in vitro stability of the ^177^Lu-DMSA@SPIONs was assessed at ambient temperature (RT) and at 37 °C in human serum and saline, as shown in Figure 3. The radiochemical purity was analyzed by radiochromatographic analysis at 2, 12, 24, 72, 96, and 144 h. The results obtained showed very high radiochemical purity and less than 2.2 ± 0.5%, 3.6 ± 0.7%, and 4.2 ± 1.0% of ^177^Lu^3+^ was released up to 144 h after keeping the original suspension at ambient temperature, and after their incubation in human serum at 37 °C, and saline solution at 37 °C, respectively. It is clear from this study that ^177^Lu-DMSA@SPIONs showed high in vitro stability up to 144 h.

### 3.3. Biodistribution of ^177^Lu-DMSA@SPIONs

**Figure 4:**
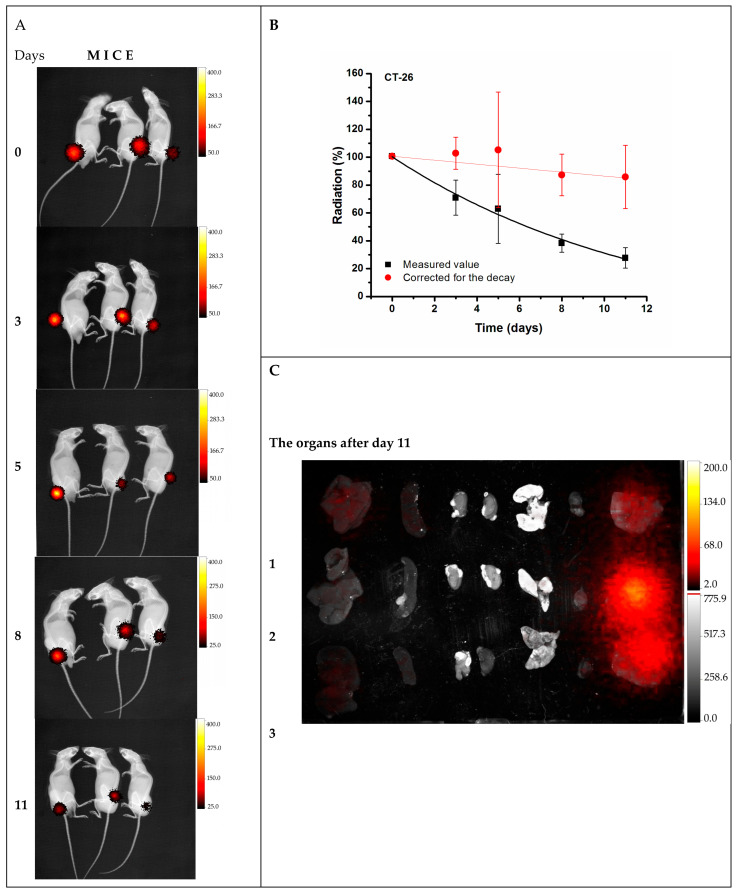
Biodistribution of ^177^Lu-DMSA@SPIONs after intratumoral injection into CT-26 tumor bearing mice; (**A**) in vivo imaging of the animals with radioactivity-to-light detection at days 0–11, (**B**) pharmacokinetic profile of the nanomaterial in tumor in vivo, obtained after an integration of the light intensities from panel (**A**); (**C**) radioactivity in the tumor and organs of interest at day 11.

Pharmacokinetic studies of ^177^Lu-DMSA@SPIONs were performed by in vivo imaging after intratumoral injection of 3.7 MBq/100 μg/50 µL of ^177^Lu-DMSA@SPIONs in CT-26 tumor-bearing mice at 0, 3, 5, 8, and 11 days after the injection (Figure 4). The injected radioactivity was apparently retained in the tumor tissue, as it can be observed in the intensity of the radiation-generated light at the place of the administration (Figure 4A). Integration of the light intensities, which are proportional to the radioactivity level when corrected for the ^177^Lu decay, shows a minimal decrease in the radioisotope level during the time course of the experiment, resulting in their long retention in the tumor tissue (>95% ID) (Figure 4B). Contrary, radioactivity was not observed in the main organs at the end of the experiment (Figure 4C). These data indicate that the intratumoral injection, due to the high exposure of tumors but low exposure of healthy organs and tissue, could be more effective yet safer than the intravenous route, making it a promising route for applications in cancer treatment.

**Figure 5:**
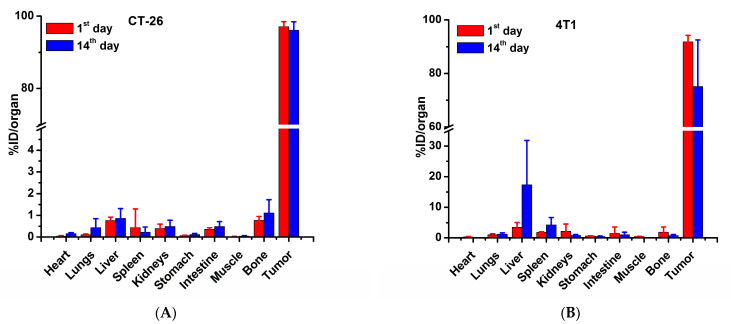
Biodistribution of ^177^Lu-DMSA@SPIONs in tumor and organs of CT-26 (**A**) and 4T1 (**B**) tumor-bearing mice after intratumoral injection of 3.7 MBq/100 μg of the nanoplatform in 50 µL vehicle. The values shown represent mean ± SD (*n* = 5–7).

In order to more precisely determine the biodistribution of the ^177^Lu-DMSA@SPIONs in the tumor tissue and organs (Figure 5), they were injected intratumorally (i.t.) into CT-26 (A) and 4T1 (B) tumor-bearing mice. The level of radiation was determined by a gamma counter in tumors and organs of interest after surgical removal on days 1 and 14. The results showed that there was high tumor radioactivity retention (90–95%ID on day 1 after the injection for both tumor types. This value remained as high as 92%ID for CT-26 but dropped down to ~72%ID for 4T1 on day 14. All the organs showed radioactivity of less than 1%ID through the whole observation period except the liver of 4T1 mice, where the radioactivity increased from 3.2%ID on day 1 to 19.8%ID on day 14, and spleen radioactivity, which increased from 1.5%ID at day 1 to 4.3%ID on day 14.

We assume that these differences in the retention of ^177^Lu-DMSA@SPIONs in the CT-26 and 4T1 tumors were caused by the structure of the tumors themselves. As CT-26 tumor has a consistent structure and was poorly vascularized, the leakage of radioactivity from them was limited. Unlike CT-26 tumors, the leakage of radioactivity from 4T1 was more significant. It may be explained by the more aggressive growth of 4T1 tumors and, thus, via their large vascularization, more of the injected nanomaterial may get removed by this route. However, knowing that the tumors frequently possess a stroma whose integrity to a great degree affects the transfer of material in and out of the tumor, it is possible that the stroma difference between those two tumors plays a role in the observed difference regarding the nanomaterial leakage [36,37].

### 3.4. The Therapeutic Efficacy of ^177^Lu-DMSA@SPIONs

To improve the understanding of the biological behavior of ^177^Lu-DMSA@SPIONs in tumors after intratumoral injection, the influence of the type of the tumor on the diffusion of nanoparticles from the injection site should be considered. Ideally, radiolabeled nanoparticles, after local injection into tumor tissue, should spread evenly from the injection site throughout the tumor mass. Addressing this issue is important because if the particles remain only at the site of application, the effect of radiation on distant parts of the tumor, depending on its tissue penetration range, may be insufficient, which calls into question the therapeutic effect of radiolabeled nanoparticles, especially in larger tumors. On the other hand, a well localized accumulation may be beneficial by allowing precise location of the radiotherapeutic, limiting exposure to the healthy tissue.

The therapeutic efficacy of ^177^Lu-DMSA@SPIONs was determined by estimating the tumor volume in CT-26 and 4T1 tumor-bearing mice up to 14 days after single or repeated intratumoral injection, respectively. At the end of the follow-up, the mice were sacrificed, and the tumors were surgically removed to measure their mass. The correlation between the volumes and masses is given in the Supplementary Materials. Considering that the tumor volume is estimated starting with a presumption that the tumors are close to a spherical shape (which frequently is not the case) and that the morphology of the tumor may obscure its true size, the final excised tumor masses are far better indicators of the therapeutic effect. 

**Figure 6:**
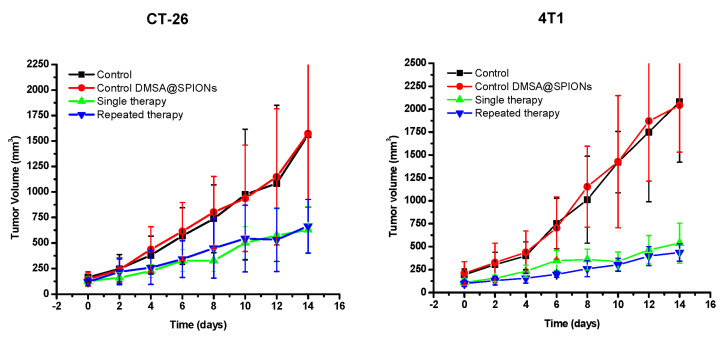
Tumor growth curves of CT-26 and 4T1 xenografts on BALB/c mice after intratumoral therapy with ^177^Lu-DMSA@SPIONs at 3.70 MBq by a single (day 0) or repeated (days 0 and 5) administration. The values shown represent mean ± SD (*n* = 5–6).

In the mice bearing CT-26 and 4T1 tumors, the obtained results revealed similar high therapeutic efficacy of ^177^Lu-DMSA@SPIONs after the single or repeated therapy was applied, as demonstrated by suppressed tumor growth at the end of the therapy (Figure 6). In the first four days of treatment, the tumor volumes of the treated groups did not differ significantly compared to the control groups (*p* > 0.05). After the sixth day of therapy, the difference became significant (*p* < 0.05). At the end of the observation (the 14th day after injection), the average tumor volumes, as well as tumor masses of all treated groups, were smaller than those in control groups, being ~2.5 times smaller for CT-26 and ~5.5 times smaller for 4T1 tumors. We consider that the beta radiation of lutetium-177 (^177^Lu) caused impaired tumor growth in all treated groups. In both control groups, the tumor growth was similar, indicating that non-radioactive DMSA@SPIONs particles did not affect tumor growth. The obtained results of the therapeutic efficacy of ^177^Lu-DMSA@SPIONs are in accordance with the results of similar studies using ^177^Lu [38,39]. The differences in the behavior of CT-26 and 4T1 may be explained by their intrinsic differences (rate of growth, status of the ROS scavengers, DNA repair mechanism, etc.) or at the morphology level (stroma structure, vascularization, etc.), which could impair the nanoparticles retention or facilitate the leakage. 

Being implanted s.c. on immune-competent mice, another mechanism by which the radiation may cause the antitumor effect may not be by killing the tumor cells directly but rather by affecting the tumor-infiltrating immune cells. Namely, tumors are frequently an immune-protected zone, with a delicate balance of the cells with pro- and anti-tumor effects. If damaged by the radiation, they may result in infiltration of killer T-cells or allow the tumor antigen presentation to antigen-presenting cells, thus leading to effective cellular and/or humoral antitumor immunity. This effect, if proven, could be a potent strategy to treat solid tumors because it could act not only on the tumor treated but on eventual metastases and even serve as a guard for relapse of the same kind of tumor.

**Figure 7:**
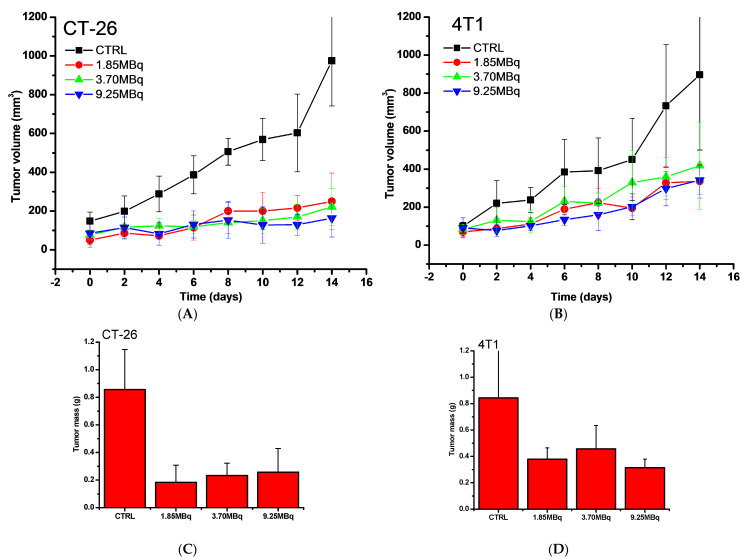
Tumor growth curves (**A**,**B**) and final tumor masses (**C**,**D**) of BALB/c mice with CT-26 (**A**,**C**) and 4T1 (**B**,**D**) xenografts intratumorally treated with ^177^Lu-DMSA@SPION by a single injection of 1.85, 3.70, and 9.25 MBq/100 μg/50 µL/100 mm^3^ of tumor. The values represent mean ± SD (*n* = 5).

To further investigate the potency of the antitumor effect and tailor the dose of radiation, the experiment was repeated on CT-26 and 4T1 tumor models, but with the dose decreased to 1.85 MBq/100 μg /50 µL of ^177^Lu-DMSA@SPIONs per 100 mm^3^ and increased to 9.25 MBq/100 μg/50 µL of ^177^Lu-DMSA@SPIONs per 100 mm^3^ of tumor (Figure 7).

As expected, all the treated groups responded well to the therapy, significantly slowing the tumor’s growth. Interestingly, ^177^Lu-DMSA@SPIONs indeed efficiently suppressed the tumor growth even at a dose as low as 1.85MBq, showing a remarkable therapeutic effect. Moreover, the effect of the five times increased dose did not increase the effect significantly. It indicates that the tumor’s response to the irradiation may be of a sigmoidal rather than linear nature, where the dose of 1.85MBq may be already close to the plateau of the effect. Another possibility is that being retained just locally and possessing low-range penetration, because the reaching zone was already sufficiently irradiated, the therapy cannot further benefit from a higher level of radiation. 

These findings support the application of ^177^Lu-bound nanoparticles in NBT, promising low levels of exposure to the healthy organs, so no general or tissue-specific toxicity is expected. Another way of application that may benefit from the short distance penetration of ^177^Lu would be to apply the material as an array of spots rather than a single deposit dose.

Regarding the body weights of both CT-26 and 4T1 tumor-bearing mice, no statistically significant differences were observed between the control and treatment groups during the study period, regardless of the number or the therapies nor the level of the doses. Hence, we can assume that intratumorally injected ^177^Lu-DMSA@SPIONs do not have a significant general toxic effect.

### 3.5. Histopathology Analysis of Tumors and Organs after Intratumoral Injection of ^177^Lu-DMSA@SPIONs in CT-26 and 4T1 Tumors Bearing Mice

**Figure 8:**
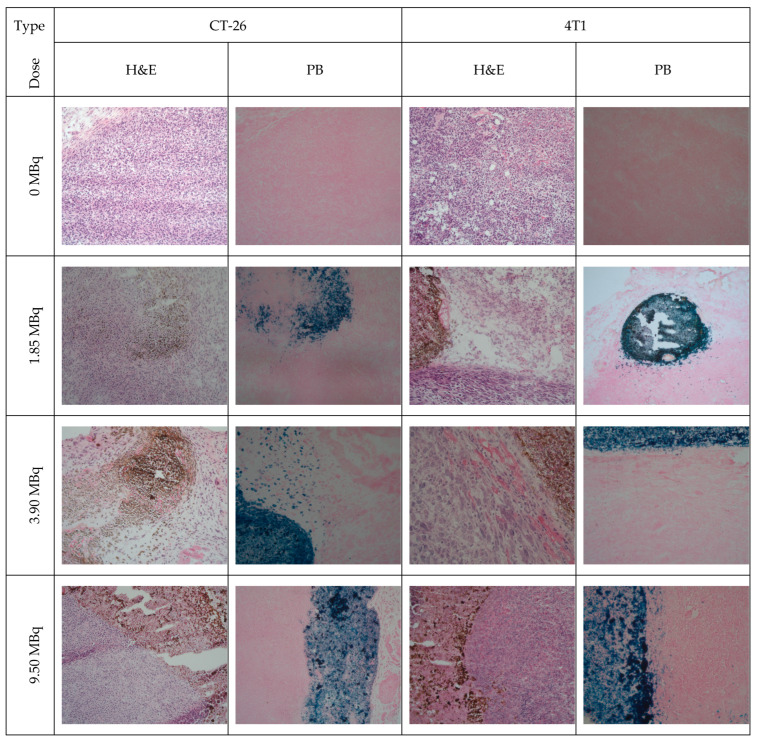
Photomicrographs of CT-26 and 4T1 tumor tissue after single dose of 1.85, 3.70, and 9.25 MBq/100 μg/50 μL per 100 mm^3^ tumor volume (H&E and PB staining, 200× magnification).

CT26 and 4T1 tumor models implanted subcutaneously in mice were used to observe the intratumoral distribution and the effect on the tumor cells of ^177^Lu-DMSA@SPIONs applied as nanobrachytherapy. For that purpose, a routine H&E staining was used to demonstrate tumor morphology and necrosis as the main secondary change, as well as the effect on the cell level, while a Prussian blue stain was used to demonstrate the nanomaterial distribution (Figure 8 and Supplementary Materials). Further, the liver and kidneys of the control and treated animals were prepared and analyzed in the same way to determine eventual signs of toxicity (Supplementary Materials).

The tumor growth was significantly hampered after intratumoral injections of ^177^Lu-DMSA@SPIONs at all the doses applied when compared with the control groups. The tumor tissue showed moderate necrosis, especially at the injection site. Interestingly, in both tumor types, it was found that the applied nanomaterial was retained and localized mostly at the place of injection. The lateral section revealed an almost perfect circle of accumulated nanomaterial (blue color on PB-stained and brown color on H&E-stained slides). The signs of mainly moderate necrosis were found in the center of the zone with necrotic cells, mostly represented as the nucleus shrinks and the chromatin condenses. It indicates that the needle-penetrating path was probably unhealed due to the presence of radiation, even though the section was made 14 days after the therapy. A similar observation was frequently present in longitudinal sections of the injection place, showing an almost linear deposition of the blue material. In most of the slides, just a very limited number of nanoparticles was observed bordering the place of the injection. This appearance was more abundant in 4T1 tumors, while in CT-26 tumors, some degree of nanomaterial spreading was noticed on a number of slides. It demonstrates that the ^177^Lu-DMSA@SPIONs very well mimic a “needle” used in classic nanobrachytherapy, further straightening the potential of this material. 

Histopathological examination revealed that the CT-26 tumor tissue showed a dense structure and homogeneously dispersed cancer polymorphic cells with round to oval nuclei and slightly basophilic cytoplasm around them, as well as poor vascularization (Figure 8).

Although the histopathological analysis reveals that the particles are mainly located at the injection site, while a smaller number is in the surrounding tissue, the radiation from lutetium-177 leads to significant regression of the entire tumor (fields of moderate to abundant necrosis). It is assumed that this is related to the structure of the tumor itself (a dense structure and poor vascularization). 

Histopathology analysis of ^177^Lu-DMSA@SPIONs treated 4T1 tumors revealed that, based on the obtained results, the nature of 4T1 tumor (highly tumorigenic and invasive, very similar to advanced human stage IV breast cancer) [40,41] radiation properties lutetium-177 as well as intratumoral spread of radiolabeled nanoparticles have a significant impact on the therapeutic effect of ^177^Lu-DMSA@SPIONs regardless the dose applied.

The analysis of the liver and kidney sections of the control and treated animals revealed just a slight sign of toxicity, not expecting to have a significant effect on the organ function or animals’ health. The detailed analysis is given in Supplementary Materials and Figure S6.

Overall, we demonstrated that intratumoral application of radionuclide-labeled nanomaterial has indeed a great potential for the therapy of solid tumors, possessing great efficiency and lacking the systemic toxicity. However, tumor properties such as the tumor type, the stroma structure, the growth rate, etc., are important parameters to consider for proper choice of the radionuclide used. Moreover, we have shown that the uniform intratumoral distribution of particles is difficult to achieve even with direct intratumoral injection, let alone intravenous administration. Such findings confirm the need for personalized nanotherapy, i.e., for carefully selecting patients for a particular type of nanotherapy to increase therapeutic efficacy even if nanoparticles are applied by direct intratumoral injection. It is also very important to start the treatment at an early stage of the tumor when the size of the tumor is smaller and before any local or distant metastatic spread.

## 4. Conclusions

In summary, the synthesized DMSA@SPIONs are shown to be stable, non-toxic, and suitable for the radiolabeling by different radionuclides. The aforementioned nanoparticles were radiolabeled with ^177^Lu in a high yield (>86%), showing high in vitro stability in saline and human serum for 6 days (>95%). The results of ex vivo biodistribution of ^177^Lu-DMSA@SPIONs after i.t. application in CT-26 and 4T1 mouse tumor xenografts model showed high i.t. retention with minimal leakage in both tumor xenografts model for 14 days. After the therapy, all the treated groups of CT-26 and 4T1 mouse tumor xenografts responded well to the therapy, significantly slowing the tumor growth, with minimal radiation exposure to normal organs and without signs of general toxicity. The therapy was highly effective even at a dose as low as 1.85 MBq, which could be important from the point of view of protecting the surrounding healthy organs. The results obtained are encouraging for the potential application of ^177^Lu-DMSA@SPIONs for localized cancer therapy of humans in a nanobrachytherapy approach; therefore, we believe ^177^Lu-DMSA@SPIONs deserve further research toward that goal.

## Data Availability

The data presented in this study are available in this article and Supplementary Materials.

## Supplementary Materials

The following supporting information can be downloaded at: https://www.mdpi.com/article/10.3390/pharmaceutics15071943/s1, Figure S1: Individual tumors growth for CT-26 mouse tumors; Figure S2: Individual tumors growth for 4T1 mouse tumors; Figure S3: Body masses of mice bearing CT-26 or 4T1 tumor treated by single or repeated therapy approach as shown in Figure 1 and Figure 2; Figure S4: Individual tumors growth for CT-26 and 4T1 mouse tumors treated by 1.85, 3.70 and 9.25 MBq/200 µg/50 µL/100 mm^3^ tumor; Figure S5: Histopathology of CT-26 and 4T1 tumors from Figure 8, magnification 400 times. Figure S6: Histopathology of liver and kidneys of control and treated animals.
