# Supplementary material for: Synthesis, Characterization, and Therapeutic Efficacy of 177Lu-DMSA@SPIONs in Nanobrachytherapy of Solid Tumors
Source: Pharmaceutics. 2023 Jul 13;15(7):1943. doi: 10.3390/pharmaceutics15071943 (PMC10384743; doi:10.3390/pharmaceutics15071943)
Supplement: Supplementary file 1 [file pharmaceutics-15-01943-s001.zip › pharmaceutics-2488002-supplementary.pdf]

Supplementary materials

Synthesis, Characterization, and Therapeutic Efficacy of ^{177}Lu-DMSA@SPIONs in Nanobrachytherapy of Solid Tumors

Dragana Stanković [1], Magdalena Radović [1], Aljoša Stanković [2], Marija Mirković [1], Aleksandar Vukadinović [1], Milica Mijović [3], Zorana Milanović [1], Miloš Ognjanović [1], Drina Janković [1], Bratislav Antić [1], Sanja Vranješ-Đurić [1], Miroslav Savić [4] and Željko Prijović [1,*]

[1] Vinča Institute of Nuclear Sciences—National Institute of the Republic of Serbia, University of Belgrade, 11001 Belgrade, Serbia; dragana.s@vin.bg.ac.rs (D.S.); magdalena.lazarevic@vin.bg.ac.rs (M.R.); mmarija@vin.bg.ac.rs (M.M.); vukadinovic@vin.bg.ac.rs (A.V.); zorana.milanovic@vin.bg.ac.rs (Z.M.); miloso@vin.bg.ac.rs (M.O.); drinaj@vin.bg.ac.rs (D.J.); bantic@vin.bg.ac.rs (B.A.); sanjav@vin.bg.ac.rs (S.V.-Đ.)

[2] University Clinical Centre of the Republic of Srpska, 78000 Banja Luka, Bosnia and Herzegovina; aljosa.stankovic@kc-bl.com

[3] Faculty of Medicine, Institute of Pathology, University of Priština in Kosovska Mitrovica, 38220 Kosovska Mitrovica, Serbia; milica.mijovic@med.pr.ac.rs

[4] Faculty of Pharmacy, University of Belgrade, 11000 Belgrade, Serbia; miroslav@pharmacy.bg.ac.rs

* Correspondence: zprijovic@vin.bg.ac.rs

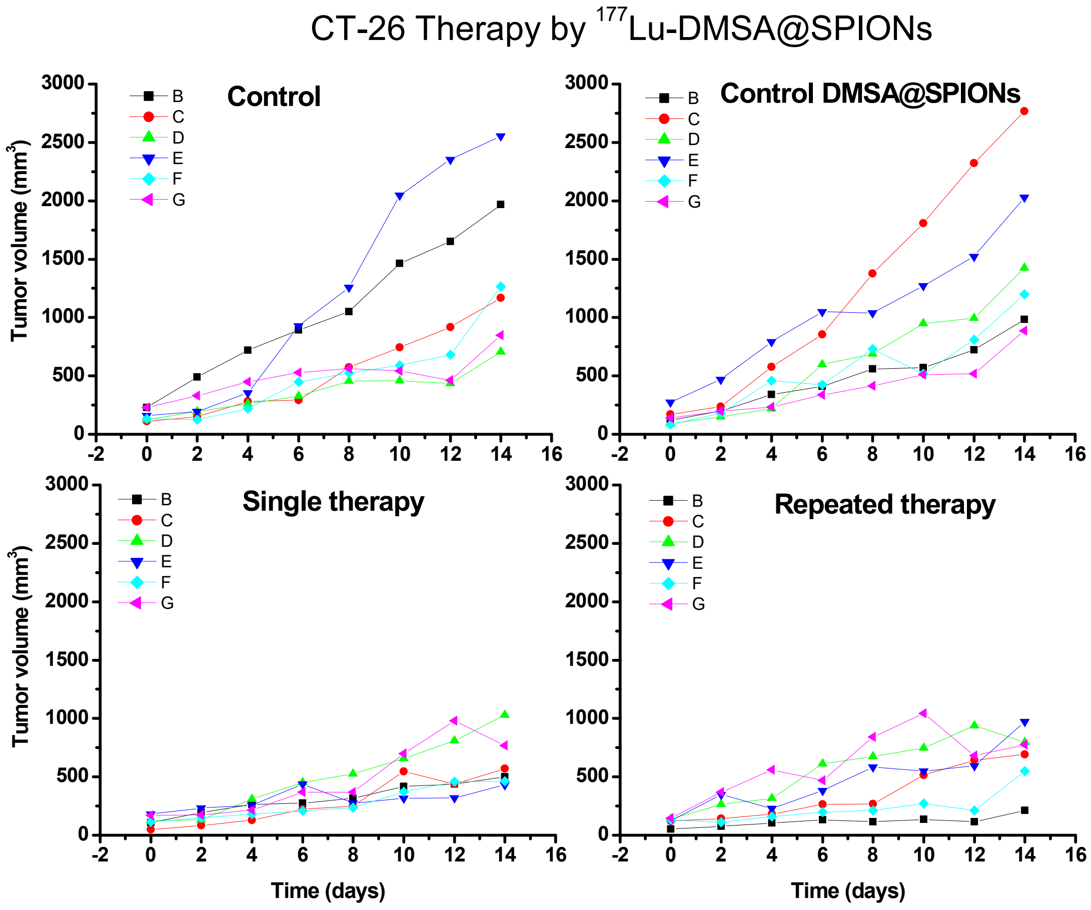

Figure S1. Individual tumors growth for CT-26 mouse tumors: untreated control (A), treated by non-labeled SPIONs@DMSA (B), injected by 3.70 MBq/50µL/100 mm^3 of tumor once at day 0 (C) or twice at days 0 and 5 (D).

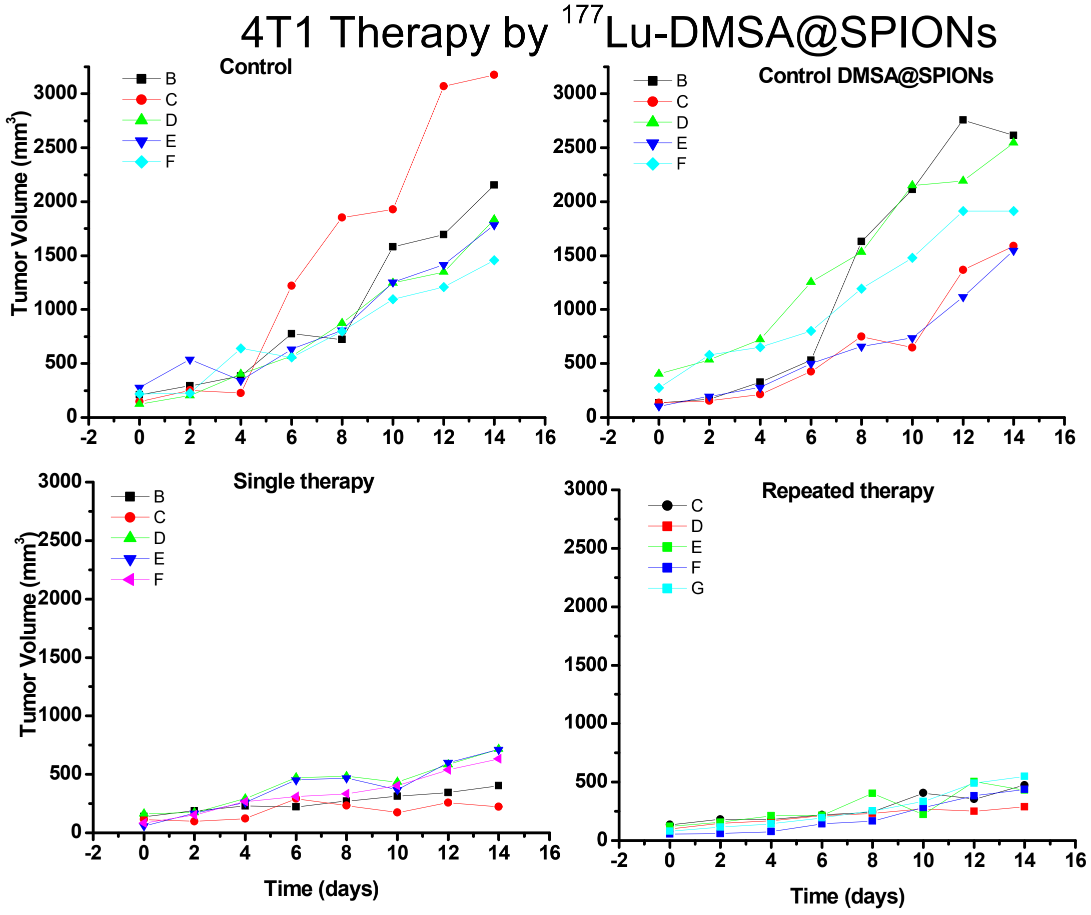

Figure S2. Individual tumors growth for 4T1 mouse tumors: untreated control (A), treated by non-labeled SPIONs@DMSA (B), injected by 3.70 MBq/50µL/100 mm³ of tumor once at day 0 (C) or twice at days 0 and 5 (D).

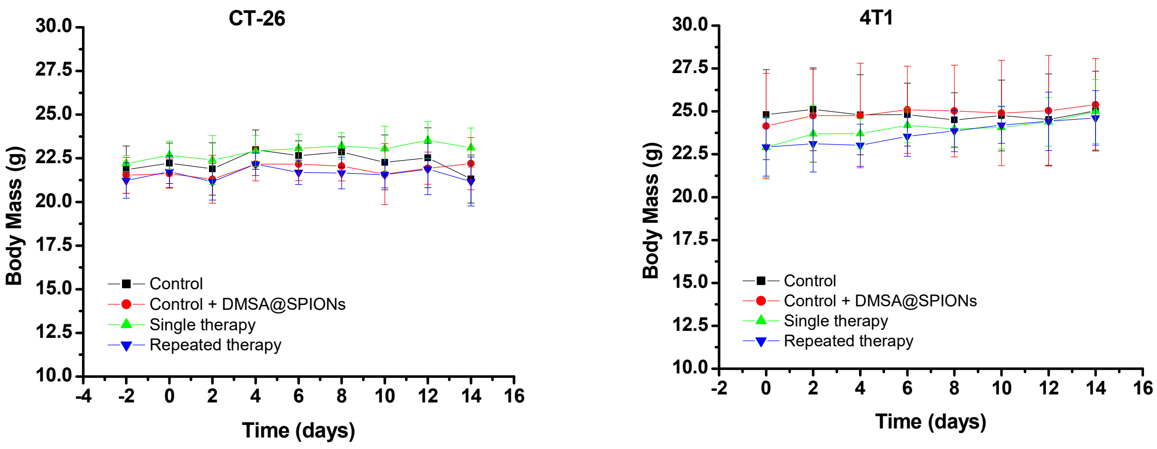

Figure S3. Body masses of mice bearing CT-26 or 4T1 tumor treated by single or repeated therapy approach as shown in Figure S1 and Figure S2.

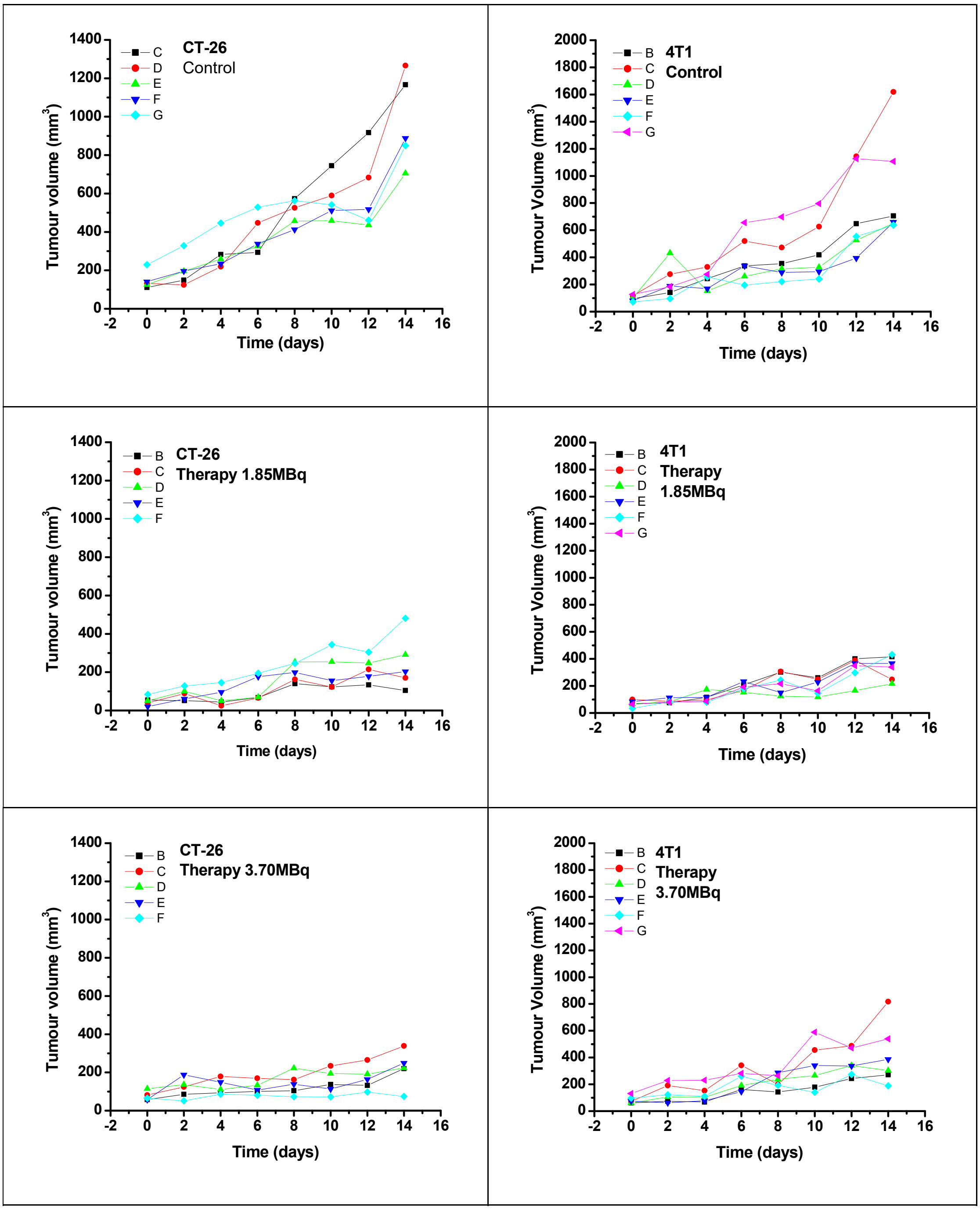
CT-26
Control
Tumour volume (mm3)
Time (days)
C
D
E
F
G
4T1
Control
Tumour Volume (mm3)
B
CT-26
Therapy 1.85MBq
4T1
Therapy
1.85MBq
CT-26
Therapy 3.70MBq
4T1
Therapy
3.70MBq

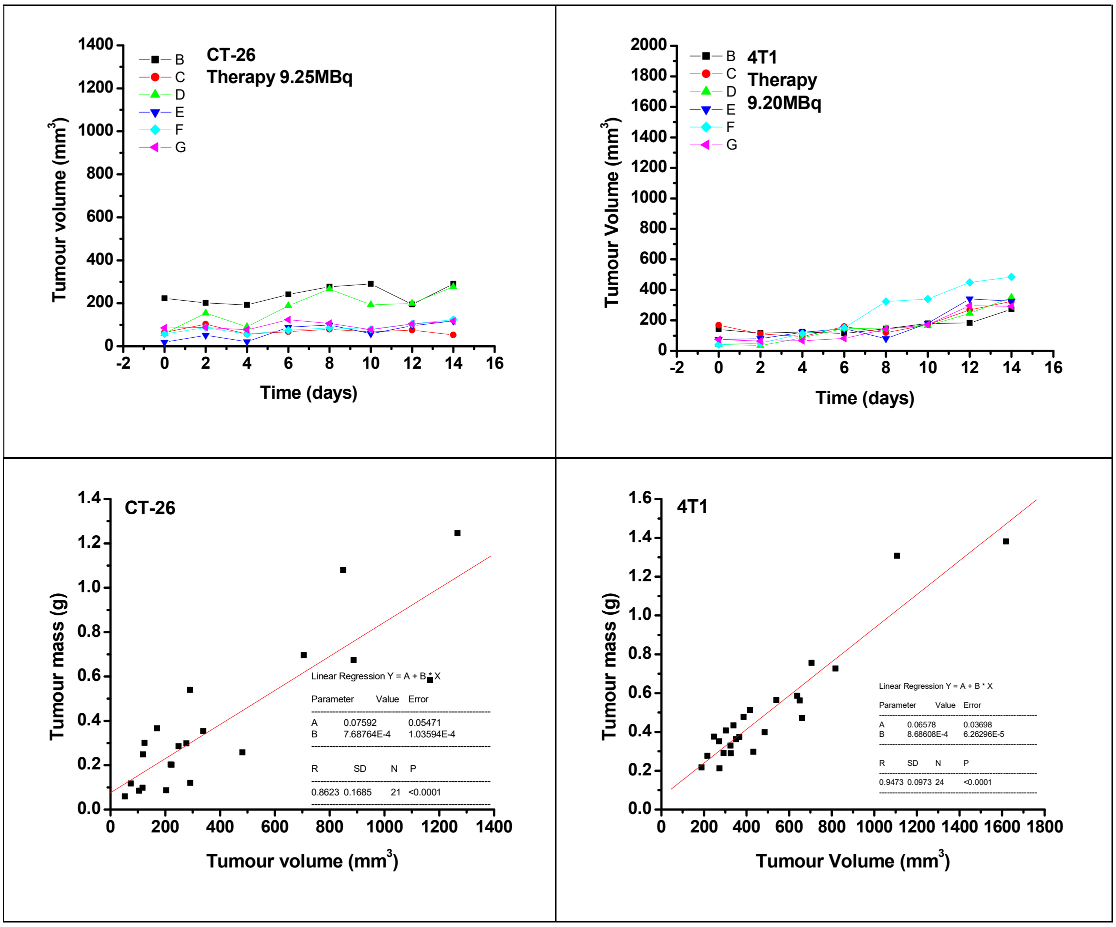

Figure S4. Individual tumors growth for CT-26 and 4T1 mouse tumors treated by 1.85, 3.70 and 9.25 MBq/200µg/50µl/100 mm^3 tumor: untreated control (A,F), treated by ^{177}Lu-SPIONs@DMSA at 1.85 MBq (B,G), 3.70 MBq (C,H) or 9.25 MBq (D,I) dose. The correlation of the estimated tumor volumes and excised tumor masses at day 14th(E,J).

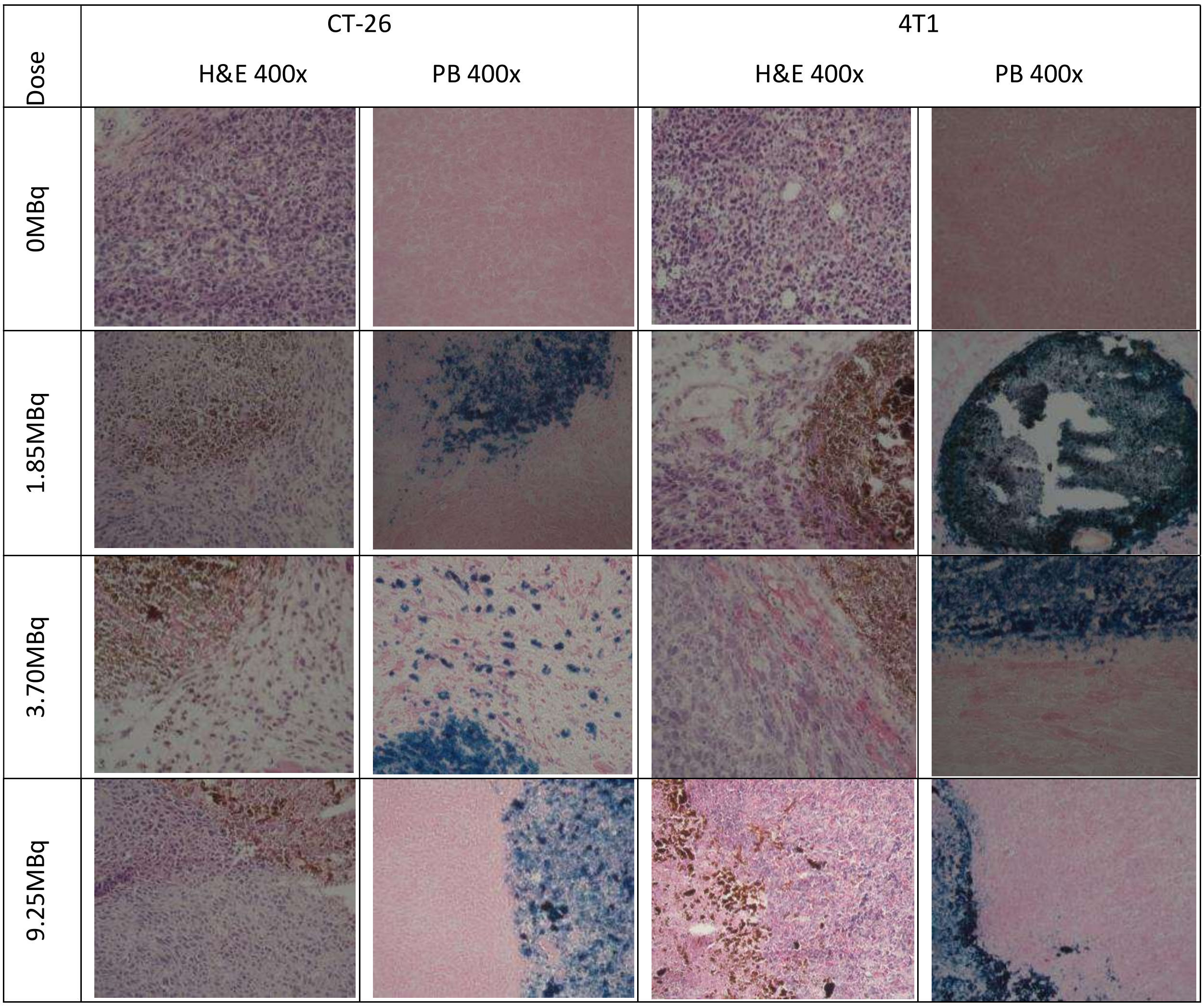

Figure S5. Histopathology of CT-26 and 4T1 tumors from Fig.8, magnification 400-times.

Histo-pathological analysis of liver and kidney of control and treated animals

We investigated histo-pathological patterns of liver according to specific type of hepatotoxicity lesion:

1. zonal degeneration/necrosis,
2. cholestasis (inflammation - cholestatic hepatitis or it can be bland - without any parenchymal inflammation),
3. non-specific hepatitis (scattered foci of cell necrosis may accompany lymphocytic infiltration),

4. steatosis (hepatotoxicity may manifest as triglyceride accumulation, which leads to either small-droplet (microvesicular) or large-droplet (macrovesicular) fatty liver), as well as
5. vascular lesions (especially venooclusive disease that results from injury to the vascular endothelium).
Results: There were no histo-pathological signs of hepatotoxicity in CT-26 animals, however the very slight microvesicular steatosis was found in 4T1 animals.

Kidney damages induced by nephrotoxicants consideration between acute kidney injury (AKI) and chronic kidney disease (CKD), both in terms of the rate of functional decline and the length of time that renal function is decreased.
Histo-pathological patterns of kidney damage include changes to the tubules, glomeruli, the interstitium and the intra-renal blood vessels. They can represent as:
1. parenchymatous degeneration of the epithelial cells of proximal tubules,
2. cell death and loss of the brush border membrane and polarity,
3. proximal tubule necrosis and
4. vascular lesions.
Proximal tubules are the primary target of a majority of nephrotoxicants.
Results: We only found parenchymatous degeneration of the epithelial cells of proximal tubules as slight changes in 4T1 animals and moderate changes in CT-26 animals.

	CT-26		4T1	
	200x	400x	200x	400x
Liver	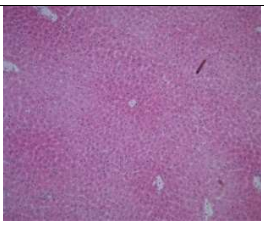	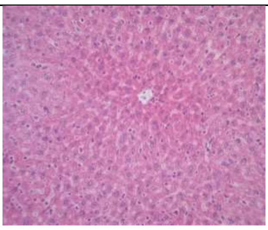	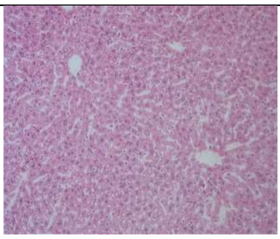	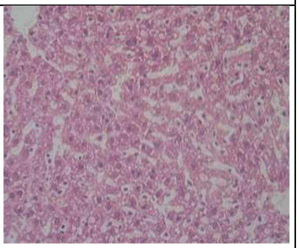
Kidneys	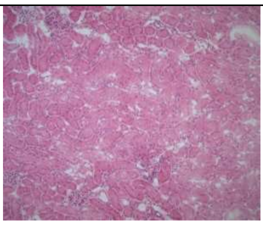	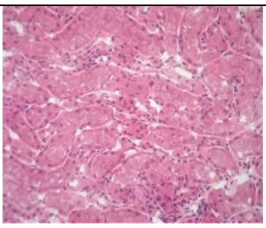	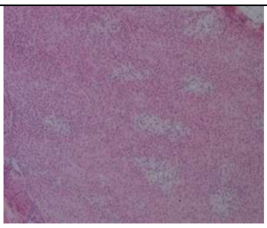	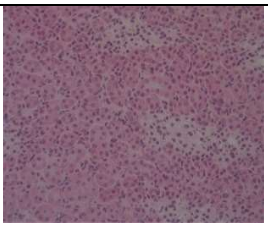

Figure S6: Histo-pathological analysis of liver and kidney of control and treated animals
